# Diagnostic Accuracy of Computed Tomography for the Prediction of the Need for Laparotomy for Traumatic Hollow Viscus Injury: Systematic Review and Meta-Analysis

**DOI:** 10.3390/jpm11121269

**Published:** 2021-12-01

**Authors:** Cheng-Chieh Hsia, Chen-Yu Wang, Jen-Fu Huang, Chih-Po Hsu, Ling-Wei Kuo, Chun-Hsiung Ouyang, Chien-Hung Liao, Huan-Wu Chen

**Affiliations:** 1 Department of Traumatology and Emergency Surgery, Chang Gung Memorial Hospital, Chang j-F Gung University, Taoyuan 33328, Taiwan; capriole823@gmail.com (C.-C.H.); wanglayla8151@gmail.com (C.-Y.W.); jenfu0211@yahoo.com.tw (J.-F.H.); m7831@cgmh.org.tw (C.-P.H.); m0102@cgmh.org.tw (L.-W.K.); detv090@gmail.com (C.-H.O.); 2 Department of Surgery, Keelung Chang Gung Memorial Hospital, Chang Gung University, Keelung 20401, Taiwan; 3 Department of Surgery, Linkou Chang Gung Memorial Hospital, Chang Gung University, Taoyuan 33305, Taiwan; 4 Department of Medical Imaging & Intervention, Chang Gung Memorial Hospital, Chang Gung University, College of Medicine 5 Fu-Hsing Street, Gueishan, Taoyuan 33305, Taiwan

**Keywords:** meta-analysis, systematic review, traumatic bowel injury, traumatic hollow viscus injury, computed tomography, diagnostic accuracy

## Abstract

Background: Traumatic hollow viscus injury (THVI) is one of the most difficult challenges in the trauma setting. Computed tomography (CT) is the most common modality used to diagnose THVI; however, various performance outcomes of CT have been reported. We conducted a systematic review and meta-analysis to analyze how precise and reliable CT is as a tool for the assessment of THVI. Method: A systematic review and meta-analysis were conducted on studies on the use of CT to diagnose THVI. Publications were retrieved by performing structured searches in databases, review articles and major textbooks. For the statistical analysis, summary receiver operating characteristic (SROC) curves were constructed using hierarchical models. Results: Sixteen studies enrolling 12,514 patients were eligible for the final analysis. The summary sensitivity and specificity of CT for the diagnosis of THVI were 0.678 (95% CI: 0.501–0.809) and 0.969 (95% CI: 0.920–0.989), respectively. The summary false positive rate was 0.031 (95% CI 0.011–0.071). Conclusion: In this meta-analysis, we found that CT had indeterminate sensitivity and excellent specificity for the diagnosis of THVI.

## 1. Introduction

Traumatic hollow viscus injury (THVI) is a difficult challenge in the trauma setting. THVI can induce active bleeding from disrupted mesenteric vessels or cause bowel discontinuity. Furthermore, discontinuity of the gastrointestinal (GI) tract causes spillage of the bowel contents, bacterial contamination, and a predisposition to sepsis [1,2]. Unlike other viscus injuries, delayed necrosis and ischemic changes might cause permeability and discontinuity of the bowel long after the time the injury occurred, leading to a dismal prognosis. Hollow viscus injury injuries after blunt trauma are uncommon but dangerous; they are reported in 1~3% of patients with abdominal injuries. The physical presentation and examination results related to these injuries can be subtle and are often overshadowed by other injuries, resulting in a clinical diagnostic dilemma. Unrecognized bowel injuries can lead to high morbidity and mortality rates and prolonged hospital stays [3,4,5]. On the other hand, the performance of nontherapeutic surgical interventions is associated with increased morbidity and mortality and a prolonged hospital stay [6,7,8]. This dilemma makes the management of THVI even more challenging.

Computed tomography (CT) is the first choice of an advanced evaluation tool for abdominal trauma [9,10]. CT is a useful means of diagnosing THVI. Abdominal CT in a patient with a bowel injury can show the presence of free fluid, free air, GI wall thickening, GI wall discontinuity, contrast extravasation, and mesenteric streaking [8,11]. However, the performance of CT for the diagnosis of THVI has been reported to range from 11% to 98% [12,13,14,15,16].

In this study, we reviewed the available studies published in English to provide a thorough evaluation of the performance of CT with regard to the diagnosis of THVI. A meta-analysis was performed to highlight the strengths and weaknesses of emergency CT for the assessment of THVI.

## 2. Materials and Methods

The systematic literature search was performed based on the 2019 Preferred Reporting Items for Systematic Reviews and Meta-analyses (PRISMA) statement.

### 2.1. Eligibility Criteria

A study was considered eligible if it evaluated CT as the only test used to diagnose THVI and determine the need for surgery in patients presenting to the ED with blunt-force trauma and presented the surgical findings with regard to the accuracy of that diagnosis.

### 2.2. Information Sources and Search

We tried to identify all published studies that reported the accuracy of CT for the diagnosis of TVHI due to blunt-force trauma. We searched the MEDLINE and EMBASE electronic databases. The search strategy restricted the language to English and the publication dates to between January 2000 and December 2019. The MEDLINE, EMBASE, Web of Science and Cochrane Library databases were searched using the following subject headings: “computed tomography”, “hollow viscus injury”, “intestinal trauma”, “intestinal injury”, “bowel injury”, “bowel trauma”, “free air”, “pneumoperitoneum”, “free fluid”, and “hemoperitoneum”. The details are provided in the Supplementary Material. The bibliographies of relevant articles were also examined to identify other eligible studies. A THVI is defined as an injury to a hollow abdominal organ involving direct injury of the organ due to the trauma that needs resection, repair, or bleeding control as the definitive treatment or that is identified on CT and managed conservatively. We supplemented our search by manually reviewing the reference lists of all retrieved articles to identify other potentially relevant citations.

### 2.3. Study Selection

Two independent reviewers (CC Hsia and CY Wang) independently screened the titles, abstracts, and, if there was insufficient information in the abstract, full-text publications to determine the suitability of the studies for inclusion in the analysis. Studies evaluating the performance of CT for the diagnosis of blunt-force THVI were eligible if they provided the data that could be used to calculate the sensitivity and specificity for the diagnosis of blunt-force THVI. No study was excluded based on the quality of the reference standard. Case reports, editorials, abstracts, conference proceedings, studies involving military patients and studies involving patients aged <18 years were excluded.

### 2.4. Data Collection Process and Quality Assessment

Two independent reviewers (CC Hsia and CY Wang) independently extracted the study and patient characteristics, and the diagnostic accuracy of CT. Cohen’s kappa coefficient (κ) was calculated to assess the agreement between the review authors. No attempts to mask the authorship, journal name or institution were made here or in any other step of the review process. Any differences in opinion regarding inclusion were discussed with a third reviewer (CH Liao). Information about CT findings, surgical findings, true positives, true negatives, false positives, false negatives and study designs was collected. The Quality Assessment of Diagnostic Accuracy Studies 2 (QUADAS-2) checklist was used by two reviewers to assess the quality of the included studies. Publication bias was evaluated by assessing the asymmetry in Deek’s funnel plot for the weighted regression with multiplicative dispersion.

### 2.5. Diagnostic Accuracy Measures

The primary objective of this systematic review was to assess the sensitivity and specificity of CT for the diagnosis of the need for surgical intervention in THVI patients. The accuracy of a diagnostic test is assessed by calculating the sum of all true-positive and true-negative findings divided by the sample size. The summary sensitivity and specificity, with 95% confidence intervals (95% CIs), and summary receiver operating characteristic (SROC) curves were generated using hierarchical SROC models.

### 2.6. Statistical Software

The statistical analyses were performed with Review Manager software, version 5.3 (the Nordic Cochrane Centre, the Cochrane Collaboration, Copenhagen, Denmark, 2014). The SROC parameters were determined with MetaDTA: Diagnostic Test Accuracy Meta-Analysis version 1.45 [17] and R (version 4.05).

## 3. Results

**Figure 1:**
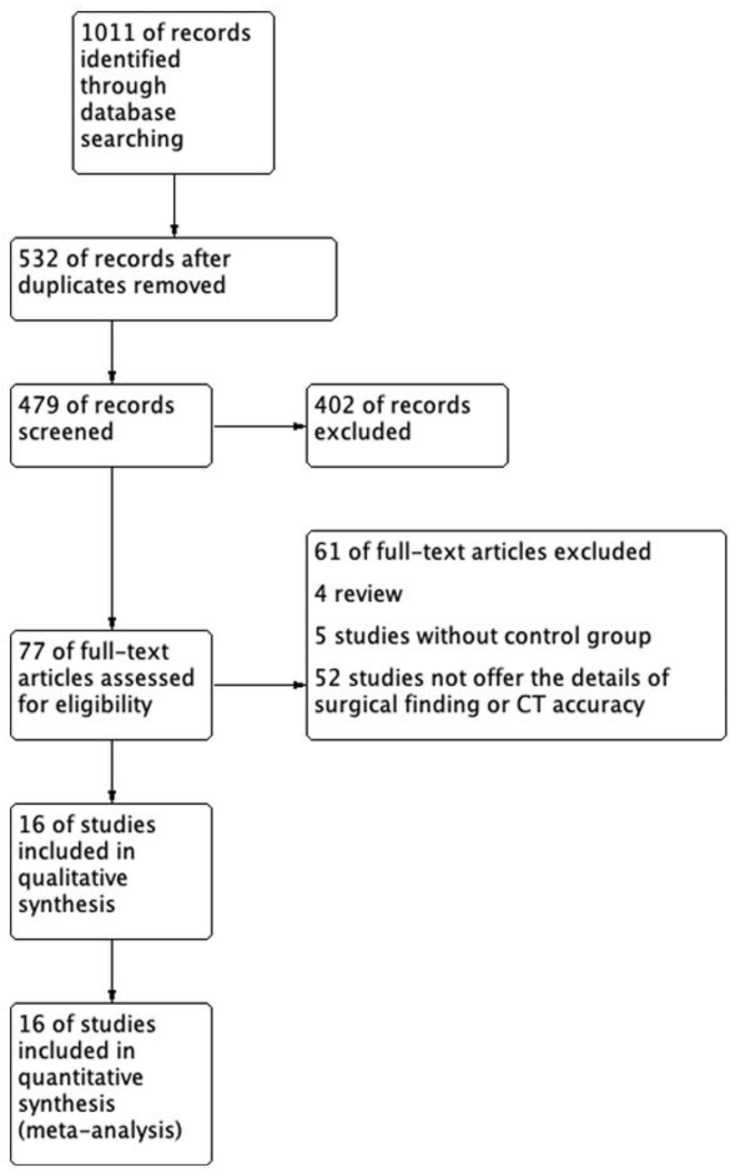
The flow diagram of this systematic review.

We identified 845 potentially relevant studies from MEDLINE and EMBASE. We excluded 366 duplicate studies and 402 studies after applying the inclusion and exclusion criteria during title and abstract screening. Finally, 77 articles were included in the full-text review, and 61 articles were excluded because of a lack of detailed CT data or inclusion of different study populations. The sixteen studies listed in Table 1 fulfilled the inclusion criteria [12,14,15,16,18,19,20,21,22,23,24,25,26,27,28,29] and were included in the qualitative analysis as Figure 1 presented.

**Figure 2:**
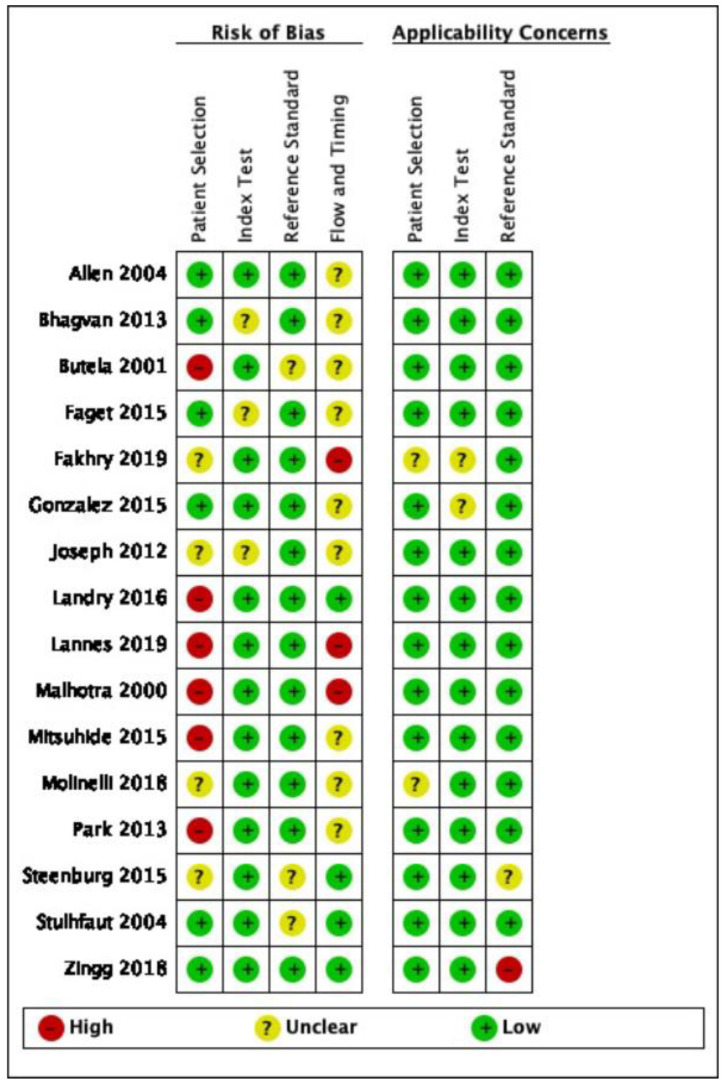
Risk of bias and applicability concerns of the included studies.

The quality assessment of the individual studies is summarized in Figure 2. No relevant applicability concerns were detected in any study. The κ coefficient for the agreement between reviewers was 0.78. A meta-analysis was performed with the SROC model.

**Figure 3:**
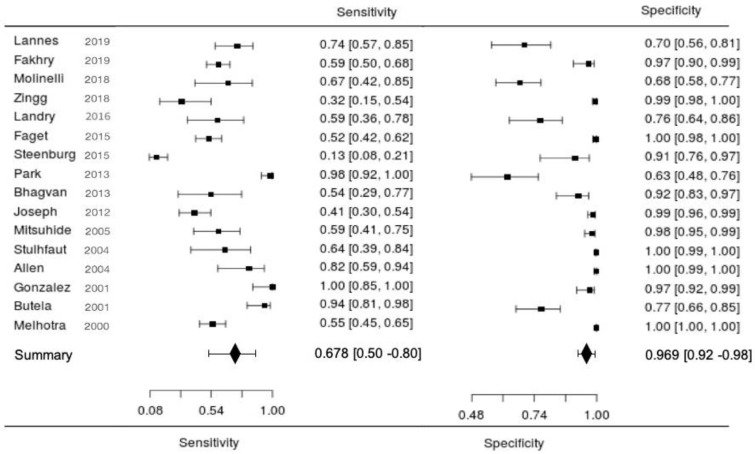
Forest plots of the sensitivity and specificity of computed tomography for the diagnosis of traumatic hollow viscus injuries.

As shown in Table 2, of the 12,514 patients who underwent CT scans, 424 patients had a true-positive diagnosis of traumatic bowel and mesenteric injury. However, a false-positive diagnosis was made in 130 patients, and a false-negative diagnosis was made in 317 patients. The sensitivity of CT for the diagnosis of THVI ranged from 13% to 95%, and the specificity ranged from 27% to 100%. The forest plot of the included studies is presented in Figure 3.

**Figure 4:**
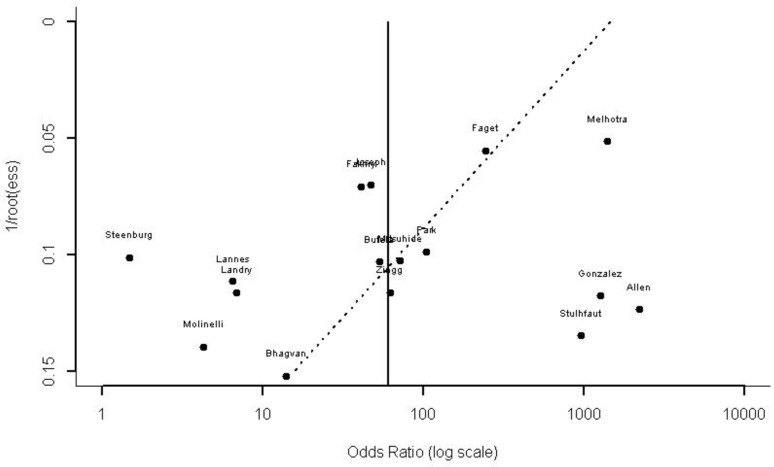
Deek’s asymmetry funnel plot for the assessment of publication bias.

Publication bias was evaluated by Deek’s funnel plot asymmetry (t = −1.8556, df = 14, *p* = 0.0847; Figure 4) and there was no asymmetry publication bias noted.

**Figure 5:**
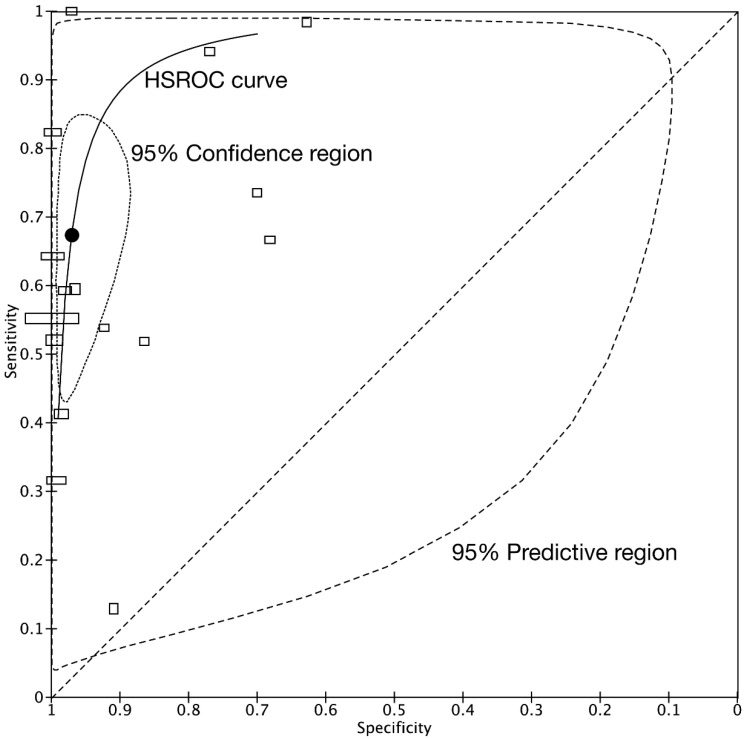
Summary receiver operating characteristic curves from all included studies. The dotted line represents the theoretical plot of a test with no discrimination ability. The summary ROC curve can be drawn through these values. The summary point estimate (black point) and its 95% confidence region are shown. The height of the rectangles is proportional to the number of patients with traumatic hollow viscus injuries across studies, and the width of the rectangles corresponds to the number of patients without traumatic hollow viscus injuries.

The bivariate model jointly synthesizes the sensitivity and specificity to give summary estimates, which are represented as the summary point on an SROC plot. Confidence and prediction regions plotted around the summary point enable joint inferences to be made about the sensitivity and specificity. The summary point for the diagnostic accuracy can be only estimated by performing a meta-analysis. This restriction reduced the studies available for inclusion in the meta-analysis from 68 to 16 studies. The summary sensitivity and specificity were 0.678 (95% CI: 0.501–0.809) and 0.969 (95% CI: 0.920–0.989), respectively. The summary false-positive rate was 0.031 (95%: CI 0.011–0.080). Figure 5 shows the summary point with a 95% confidence region and a 95% prediction region. The confidence region is based on the CI around the summary point and the available data. The prediction region around the summary point indicates the region in which we would expect results of a new study in the future to fall and is, therefore, wider than the confidence region, as it goes beyond the uncertainty in the available data.

## 4. Discussion

This systematic review summarizes the results of studies on the diagnostic performance of CT for THVIs. In this review, we found that the reported sensitivity of CT ranged from 63% to 95%, and the false-negative rate was 2.5%. However, the high specificity of CT for the diagnosis of THVI has been universally reported. The pooled analysis showed the summary sensitivity was 0.678 (0.501 to 0.809), and the summary specificity was 0.969 (0.920 to 0.989). THVIs occur in between 2% and 6% of patients with blunt-force abdominal trauma. CT is the first-line diagnostic modality for blunt-force abdominal trauma; it provides information about viscus organs that can be used to make treatment decisions. Although CT is highly specific for the diagnosis of THVI, the sensitivity is inadequate [2,27,29]. A shift has occurred towards a preference for nonsurgical management of abdominal trauma [30,31]. The presence of THVI is a crucial indicator of the need for surgical intervention [32,33], and the missed diagnosis of THVI is probably the most common cause of delayed laparotomy. A missed diagnosis leads to deferred intervention and a dismal prognosis because of uncontrolled infection and sepsis [15]. This limitation has decreased with improvements in CT technology and increased awareness of this type of trauma [23]. However, the unsatisfactory sensitivity is still a challenge [34] and is the result of two causes: incorrect interpretation of the initial images and the natural course of delayed bowel perforation. Therefore, appropriate repeated examinations and careful observation are important aspects of nonsurgical management prior to the exclusion of THVI. In this review, we found the pooled analysis showed a significant improvement in sensitivity.

The diagnostic signs and presentation on CT were also reported to be highly variable, which often led to the incorrect interpretation of the initial CT in patients with THVI [29,35]. The main characteristics of THVI include peritoneal characteristics, such as free fluid and free air; mesenteric characteristics, such as mesenteric extravasation and mesenteric stranding/hematoma; and intestinal characteristics, such as intestinal tract wall enhancement and discontinuity. The distributions of these characteristics differed based on the study, the study group, the level of experience of the examiner, and the model of CT scanner used.

Free peritoneal air was the typical presentation if there was perforation or discontinuity of the intestinal tract. However, the proportion of patients with this presentation ranged from 0% to 83% in the included studies. Intestinal tract discontinuity is another specific sign indicating THVI. A similarly large range in the proportion of patients with intestinal tract discontinuity was reported (3–100%). Free peritoneal fluid is another typical presentation of THVI on CT; this sign is common and plays an important role in the diagnosis of THVI. Several studies have shown that it is a sensitive indicator of THVI that needs surgical management; [1,36] however, several studies reported conflicting conclusions [37,38]. The lack of a typical presentation, which makes the diagnosis difficult, and the variance in the experience of the readers are important factors affecting the diagnostic accuracy. Some studies showed that the sensitivity differed based on the level of experience of the reader [6,14,25,27]. The sensitivity improved when an experienced expert read the CT scan than when a novice read the CT scan. Moreover, other authors reported that the diagnostic accuracy and sensitivity differed based on the urgency of the emergency situation in which the readers were operating [25]. To overcome this challenge, some authors have advised developing scoring systems that include imaging and clinical presentations that can be used to accurately diagnose THVI [4,16,19]. However, the results have been found to be variable and subjective.

The reason for the relatively low sensitivity of CT for the diagnosis of THVI is delayed perforation [39]. Unlike other viscus traumas, delayed ischemia, necrosis, and perforation of the bowel cannot be predicted or diagnosed on the initial CT images [31]. The challenge persists with the risk of a delayed event in which ischemia due to mesenteric injury or mural hematoma can lead to delayed perforation [40]. Repeated CT is advised to increase the diagnostic performance, [18] and the subsequent images can improve the diagnostic rate. If patients suffer from new-onset abdominal pain and peritoneal signs, additional CT scans can be performed to identify delayed THVI. Currently, there is a lack of well-designed prospective studies on the scheduling or timing of repeated CT scans. In a small group of trauma patients with questionable CT findings, a short-term (6–48 h) follow-up CT [3,18] was found to aid in confirming or excluding the diagnosis of bowel injury. Although short-term imaging follow-up may be beneficial for some patients, it may delay discharge and expose patients to unnecessary radiation. Therefore, even though several authors claim the need for and effectiveness of repeated CT, no consensus has been reached with regard to the details of these repeated examinations. Diagnostic ultrasound is another accurate method of detecting THVI [40] and can be used during follow-up. Lessons learned from battlefield medicine have shown that the use of ultrasound has the benefits of easy operation and no radiation exposure.

### Limitations

This study is a systematic review of the diagnostic accuracy of CT for THVI. All available articles were reviewed to provide an overview of current clinical practice. There were some limitations. First, we did not collect studies published in languages other than English, which might have led to the omission of relevant articles. However, all available abstracts were evaluated by our reviewers; therefore, if an article published in a language other than English had an abstract written in English, we also included those data. Second, some manuscripts were published 20 years ago, and the technology and resolution of CT at that time does not reflect the current usefulness of CT as a diagnostic tool. Third, the protocol for evaluating trauma patients has changed in the past two decades. Whole-body CT is now performed for trauma patients, whereas CT was not often performed for trauma patients in the early 2000 s. The changes in the clinical protocol and the attitude of the physicians may have led to selection bias in this review. Furthermore, because we focus on the CT diagnostic accuracy in surgical significant cases, therefore, there are several articles that cannot offer the information clearly will be not included in this review, the selection bias cannot be completely excluded. Fourth, although the studies focusing on battlefield medicine have provided valuable evidence leading to advances in the treatment of trauma, we did not include military studies in the meta-analysis because of the differences in the complexity and etiology of injuries between the military and civilian populations. Fifth, we did not search the gray literature and did not evaluate heterogeneous patient populations/settings, which might be another limitation of this review.

## 5. Conclusions

In conclusion, CT is an accurate tool for the diagnosis of THVI, with a false-negative rate of 2.5%. However, none of these signs can be used as a single predictor for delayed surgical management. To evaluate delayed THVI, well-designed prospective studies, including randomized allocation and comparison with open operations, are needed.

## Figures and Tables

**Table 1:** Characteristics of the included studies.

Author/Publication Year	Sample Period	Study Location	Ct Device; Slice Thickness	Population	Study Type
Lannes/2019 [18]	2009.3–2017.3	France	OPTIMA CT660 (GE Healthcare, Milwaukee, WI, USA); 3 mm	Single level 1 trauma left	Retrospective
Fakhry/2019 [12]	2013.10–2015.9	United states	NR; NR	Registry data	Retrospective
Molinelli/2018 [14]	2005.1–2014.10	Italy	SOMATOM Sensation 40 (Siemens Medical System, Forchheim, Germany); 1.2 mm	Single university hospital	Retrospective
Zingg/2018 [19]	2008.1–2015.6	Switzerland	Light Speed VCT 64 Pro (GE Healthcare, Milwaukee, WI, USA) 1.25 mm	Single university hospital	Retrospective
Landry/2016 [15]	2006.1–2013.6	Canada	NR; 3 mm	Single level 1 trauma left	Retrospective
Faget/2015 [16]	2004.4–2011.12	France	LightSpeed VCT 16/64 (GE Healthcare, Milwaukee, WI, USA); 3 mm	Single level 1 trauma left	Retrospective
Steenburg/2015 [20]	2007.1–2011.12	United States	64-slice MDCT (Philips Medical Systems, Andover, Mass); 4 mm	Single level 1 trauma left	Retrospective
Bhagvan/2013 [21]	2002.1–2007.12	New Zealand	High Speed Advantage (GE Healthcare, Milwaukee, WI, USA)/Siemens Volume Zoom or Sensation 16 (Siemens Medical System, Forchheim, Germany); NR.	Single Level 1 trauma left	retrospective
Park/2013 [23]	2007.1–2011.12	Korea	LightSpeed VCT (GE Healthcare, Milwaukee, WI, USA)/SOMATOM Sensation 64 or Definition AS 64 (Siemens Medical System, Forchheim, Germany); 3–5 mm	Two hospitals	Retrospective
Joseph/2012 [22]	2009.1–2011.12	United States	LightSpeed VCT (GE Healthcare, Milwaukee, WI, USA); NR	Single level 1 trauma left	Retrospective
Mitsuhide/2005 [24]	1994.4–2002.5	Japan	ProSeed Accell (GE Healthcare, Milwaukee, WI, USA); NR	Single hospital	Retrospective
Stuhlfaut/2004 [26]	2001.10–2003.9	United States	MX8000 (Philips Medical Systems, Andover, Mass); 3 mm	Single level 1 trauma left	Retrospective
Allen/2004 [25]	2000.7–2001.11	United States	CTI helical scanner (GE Healthcare, Milwaukee, WI, USA); 7 mm	Single hospital	Retrospective
Gonzalez/2001 [28]	1999.2–2000.7	United Kingdom	NR; NR	Single level 1 trauma left	Randomized controlled trial
Butela/2001 [27]	1990.6–1997.11	United States	HiLight Advantage and HiSpeed Advantage (GE Healthcare, Milwaukee, WI, USA); 7 mm	Single level 1 trauma left	Retrospective
Malhotra/2000 [29]	1995.8–1998.12	United States	SOMATOM Plus (Siemens Medical System, Forchheim, Germany); 7 mm	Single level 1 trauma left	Retrospective

**Table 2:** Summary data of the included studies with detailed data on the accuracy of computed tomography for the detection of traumatic bowel injury.

Author	N	Prevalence	True Positive	False Negative	False Positive	True Negative	Sensitivity	Specificity	Positive LR	Negative LR
Fakhry et al., 2019 [12]	203	0.57	69	47	3	84	0.595	0.966	17.25	0.41
Lannes et al., 2019 [18]	84	0.40	25	9	15	35	0.735	0.700	2.45	0.38
Molinelli et al., 2018 [14]	106	0.14	10	5	29	62	0.667	0.681	2.09	0.49
Zingg et al., 2018 [19]	698	0.03	6	13	5	674	0.316	0.993	42.88	0.69
Faget et al., 2015 [16]	553	0.17	51	47	2	453	0.52	0.996	118.39	0.48
Landry et al., 2016 [15]	72	0.31	10	7	13	42	0.588	0.764	3.82	0.56
Steenburg et al., 2015 [20]	126	0.74	12	81	3	30	0.129	0.909	1.42	0.96
Bhagvan et al., 2013 [21]	78	0.17	7	6	5	60	0.538	0.923	7.00	0.50
Park et al., 2013 [23]	106	0.59	62	1	16	27	0.984	0.628	2.65	0.03
Joseph et al., 2012 [22]	337	0.19	26	37	4	270	0.413	0.985	28.27	0.60
Mitsuhide et al., 2005 [24]	229	0.12	16	11	4	198	0.593	0.98	29.93	0.42
Allen et al., 2004 [25]	496	0.03	14	3	1	478	0.824	0.998	394.47	0.18
Stuhlfaut et al., 2004 [26]	1082	0.01	9	5	2	1066	0.643	0.998	343.29	0.36
Butela et al., 2001 [27]	112	0.30	32	2	18	60	0.941	0.769	4.08	0.08
Gonzalez et al., 2001 [28]	124	0.17	22	0	3	99	1.000	0.971	34	0
Malhotra et al., 2000 [29]	8108	0.01	53	43	7	8005	0.552	0.999	631.90	0.45
Summary	12,514	0.06	424	317	130	11,643	0.678 (0.501–0.809)	0.969 (0.920–0.989)	21.542 (8.656–57.681)	0.3322 (0.209–0.542)

N: case number, LR: likelihood ratio.

## Data Availability

The authors declare that the data supporting the findings of this study are available within the paper and its Supplementary information file.

## Supplementary Materials

The following are available online at https://www.mdpi.com/article/10.3390/jpm11121269/s1, Supplementary material included the literature review strategy and the quality assessment protocol.
