# Supplementary material for: Diagnostic Accuracy of Computed Tomography for the Prediction of the Need for Laparotomy for Traumatic Hollow Viscus Injury: Systematic Review and Meta-Analysis
Source: J Pers Med. 2021 Dec 1;11(12):1269. doi: 10.3390/jpm11121269 (PMC8708608; doi:10.3390/jpm11121269)
Supplement: Supplementary file 1 [file jpm-11-01269-s001.zip › jpm-1469663-supplementary.pdf]

Supplement 1

	Search term	Results
MEDLINE	"computed tomography"[Title/Abstract] AND ("bowel injury"[Title/Abstract] OR "bowel trauma"[Title/Abstract] OR "intestinal trauma"[Title/Abstract] OR "intestinal injury"[Title/Abstract] OR (("hollow"[All Fields] OR "hollowed"[All Fields] OR "hollows"[All Fields]) AND "viscus trauma"[Title/Abstract]) OR ((("hollow"[All Fields] OR "hollowed"[All Fields] OR "hollows"[All Fields]) AND ("viscera"[MeSH Terms] OR "viscera"[All Fields] OR "viscus"[All Fields])) AND "laceration"[Title/Abstract]) OR "intestinal laceration"[Title/Abstract] OR "free air"[Title/Abstract] OR "pneumoperitoneum"[Title/Abstract] OR "hemoperitoneum"[Title/Abstract] OR "free fluid"[Title/Abstract]	468
EMBASE	computed AND ('tomography'/exp OR tomography) AND ('bowel'/exp OR bowel OR 'intestine'/exp OR intestine) AND ('injury'/exp OR injury OR 'trauma'/exp OR trauma) AND ([article]/lim OR [article in press]/lim OR [conference paper]/lim) AND [english]/lim AND [young adult]/lim AND [humans]/lim AND [abstracts]/lim AND [2000-2019]/py<br>computed AND tomography AND free AND air AND (injury OR trauma) AND ([article]/lim OR [article in press]/lim OR [conference abstract]/lim OR [conference paper]/lim) AND [adult]/lim AND [humans]/lim AND [english]/lim AND [2000-2019]/py<br>computed AND tomography AND pneumoperitoneum AND (injury OR trauma) AND ([article]/lim OR [article in press]/lim OR [conference abstract]/lim OR [conference paper]/lim) AND [english]/lim AND	377

	[adult]/lim AND [humans]/lim AND [2000-2019]/py<br>computed AND 'tomography'/de AND free AND 'fluid'/de AND ('injury'/de OR 'trauma'/de) AND ([article]/lim OR [article in press]/lim OR [conference abstract]/lim OR [conference paper]/lim) AND [english]/lim AND [adult]/lim AND [humans]/lim AND [2000-2019]/py<br>computed AND 'tomography'/de AND 'hemoperitoneum'/de AND ('injury'/de OR 'trauma'/de) AND ([article]/lim OR [article in press]/lim OR [conference abstract]/lim OR [conference paper]/lim) AND [adult]/lim AND [humans]/lim AND [english]/lim AND [abstracts]/lim AND [2000-2019]/py	
Web of science	**TITLE:**(computed tomography) *AND* **TOPIC:**(bowel) *AND* **TOPIC:** (trauma) / **TITLE:**(computed tomography) *AND* **TOPIC:**(intestinal) *AND* **TOPIC:** (trauma) / **TITLE:**(computed tomography) *AND* **TOPIC:**(intestinal) *AND* **TOPIC:** (injury) / **TITLE:**(computed tomography) *AND* **TOPIC:**(bowel) *AND* **TOPIC:** (injury)<br>**Timespan:** 2000-2019. **Indexes:** SCI-EXPANDED.	166
Cochrane Library	computed tomography in Title Abstract Keyword AND bowel trauma in Title Abstract Keyword OR intestinal trauma in Title Abstract Keyword OR bowel injury in Title Abstract Keyword OR intestinal injury in Title Abstract Keyword	0

Supplement 2

QUADAS checklist for all diagnosis papers

No	items
1a	representative patient sample
1b	study participants clearly described
2	selection criteria clearly described
3	adequate reference standard
4	acceptable delay between tests
5	partial verification avoided
6	differential verification avoided
7	incorporation avoided
8a	adequate index test description
8b	cut-off value clearly described
9	adequate reference standard description
10	blinding for reference test results
11	blinding for index test results
12	clinical data avaiable as in practice
13	uninterpretable test results reported
14	explaining withdrawals from the study
